# Annotations

**Published:** 1903-09-12

**Authors:** 


### ANNOTATIONS.

### The Insurance of Children.

THE abuses connected with the insurance of infants and young children, though well known in certain quarters, have hardly attracted much general attention, save in the few instances where some particularly scandalous case has been brought to light. In 1896, an Act of Parliament was passed which made it illegal, *inter alia*, for any parent to receive more than £6 on the death of a child under five years of age, and also provided that an insurance society should pay only on the production of a certificate of death from the Registrar. The difficulties of proving infractions of the law in these cases are considerable, but we are glad to see that they were successfully surmounted in a case recently tried at the Bilston Police-Court. A married woman was there charged with receiving moneys from certain death clubs, and five women stewards were charged with paying moneys, without the production of the necessary death certificates. It was proved that £12 10s. from five clubs had been paid, and that three of these clubs were unregistered. It is satisfactory to know that the women were convicted and fined, and we congratulate the authorities on their vigilance. The prosecution was instituted by the Registrar of Friendly Societies.

### Vaccine Lymph and Chloroform.

THE report of the Medical Officer of the Local Government Board, just issued, contains a highly important article, by Dr. Alan Green, on the destruction of extraneous organisms in vaccine lymph, by the agency of chloroform; and the process which he describes seems likely very materially to increase the existing facilities for the performance of vaccination, and especially for meeting exceptional demands for lymph in times of panic. Our readers will be familiar with the general outline of the process by which lymph is "glycerinated," and with the manner in which, after the addition of glycerine, all the numerous extraneous organisms present with it when taken from the calf perish and disappear, leaving the "vaccine" element itself in full activity. The great objection to this process is the time required for its completion, a time so prolonged as to place great difficulties in the way of meeting any sudden or exceptional demand, and sufficient to have been put forward as an adequate reason for the refusal of the Local Government Board to undertake to supply any but authorised public vaccinators. Dr. Green has for a long time been engaged in experiments for determining the action of agents other than glycerine upon vaccine lymph, both as regards the extraneous organisms and the specific germ to which efficacy is due; and he has found that chloroform exerts a germicidal action on the extraneous bacteria of vaccine, while it has, apparently, little or no influence upon the potency of its specific germ. Moreover, it operates very rapidly, for while glycerine requires a period of about seven weeks before its purifying action is complete, chloroform, in an aqueous solution of one in 200, takes only six hours. The lymph purified by chloroform produced perfect vaccine vesicles eight months after its preparation, and, as the chloroform can be removed by evaporation from the vaccines at any time, it promises to be of the greatest service in preparing them. Dr. Green adds that, since the completion of the main body of his report, he has devised another method of treating vaccine with chloroform which, so far, has given very satisfactory results. Roughly, this method consists of passing a mixture of chloroform vapour and air through vaccines previously mixed either with distilled water or with distilled water and glycerine solution. In this way elimination of extraneous micro-organisms is found to be as complete, and in point of time more rapid than by former chloroform methods, while the specific germ remains fully active. He promises to give a further account of the method, and of the results obtained by its use, in the next report of the Medical Officer of the Board.

### The St. John Ambulance System in India.

The St. John Ambulance Association is developing into an important organisation in our Indian Empire. The aims of the Association not only include a wide extension of popular instruction in "first aid" and hygiene, but also the establishment of an organised ambulance service capable of serving under military control in the event of war. The Indian Government, whilst lending an attitude of general benevolence towards these objects, has not so far provided more active assistance, but the cause of the Association has been publicly advocated by some of the leading military authorities, including the Duke of Connaught, Lord Roberts, and Lord Kitchener, as also by many prominent Anglo-Indian and native civilians. It is argued that the potentialities of the native States in the direction of supplying trained bearer companies are very great, and that these, if properly developed, might prove as valuable, in the event of war breaking out on the North-West frontier, as did the British Red Cross Association during the recent war in South Africa. Moreover it is obvious that anything which quickens the native conscience in reference to public and private hygiene must be an influence carrying a decidedly beneficial tendency. The difficulties in this direction in India are well known to be enormous, and the absence of a sane public opinion has repeatedly paralysed the work of those concerned with the management and control of epidemic outbreaks. Thus the efforts of the St. John Ambulance Association must necessarily claim the especial sympathies of those engaged in medical work in India and of the officials more immediately responsible for the government of that vast country. It is satisfactory to find that the association recognises the value and importance of reaching the rising generation. Hence they advocate the introduction into State-aided and mission schools of instruction in first aid, and home nursing and hygiene. It is reasonable to conclude that well-planned and systematic efforts may in time succeed in producing a quiet but effective sanitary revolution in India. For this we must look to the influence of education rather than to the compulsion of authority. The crusade of the St. John Ambulance Association, therefore, deserves every encouragement and it may, we are sure, count on the good wishes of the entire medical profession.

